# Development and Optimization of Supercritical Fluid Extraction Setup Leading to Quantification of 11 Cannabinoids Derived from Medicinal Cannabis

**DOI:** 10.3390/biology10060481

**Published:** 2021-05-28

**Authors:** Sadia Qamar, Yady J. Manrique, Harendra S. Parekh, James R. Falconer

**Affiliations:** 1 Pharmacy Australia Centre of Excellence, School of Pharmacy, The University of Queensland, Brisbane, QLD 4102, Australia; y.manriqetores@uq.edu.au (Y.J.M.); h.parekh@uq.edu.au (H.S.P.); 2 School of Clinical Sciences, Queensland University of Technology, Brisbane, QLD 4000, Australia

**Keywords:** cannabis flowers, neutral cannabinoids (sp. *Sativa*), supercritical extraction, supercritical carbon dioxide (scCO_2_), SFE Nottingham unit, SFE Helix unit

## Abstract

**Simple Summary:**

This study describes the design and development of setup for the extraction of cannabis strain 1 (Cannabidiol dominant) using supercritical carbon dioxide. For this purpose, two different supercritical fluid extraction instruments were used. The extraction conditions were maintained at 37 °C and 250 bar. Different carbon dioxide inlet and outlet positions were experimented to obtain the maximum yield. A separating chamber was also designed to reduce the throttling effect and dry ice formation during the depressurization process. After developing the supercritical fluid extraction setup, ultra-high performance liquid chromatography coupled with a diode array detection quantification method for 11 cannabinoids was developed.

**Abstract:**

In this study, the optimal setup of supercritical fluid extraction (SFE) was designed and developed, leading to the quantitation of 11 distinct cannabinoids (cannabidivann (CBDV), tetrahydrocannabivann (THCV), cannabidiol (CBD), cannabigerol (CBG) cannabidiolic acid (CBDA), cannabigerolic acid (CBGA), cannabinol (CBN), delta 9-tetrahydrocannabinol (Δ^9^-THC), delta 8-tetrahydrocannabinol (Δ^8^-THC), cannabichomere (CBC) and delta 9-tetrahydrocannabinol acid (THCA-A)) extracted from the flowers of medicinal cannabis (sp. *Sativa*). Supercritical carbon dioxide (scCO_2_) extraction was performed at 37 °C, a pressure of 250 bar with the maximum theoretical density of CO_2_ (893.7 kg/m^3^), which generated the highest yield of cannabinoids from the flower-derived extract. Additionally, a cold separator (separating chamber) was used and positioned immediately after the sample containing chamber to maximize the yield. It was also found that successive washing of the extract with fresh scCO_2_ further increased yields. Ultra-high performance liquid chromatography coupled with DAD (uHPLC-DAD) was used to develop a method for the quantification of 11 cannabinoids. The C18 stationary phase was used in conjunction with a two solvent system gradient program resulting in the acquisition of the well-resolved chromatogram over a timespan of 32 min. The accuracy and precision of isolated cannabinoids across inter-and intra-day periods were within acceptable limits (<±15%). The assay was also fully validated and deemed sensitive from linearity, LOQ, and LOD perspective. The findings of this body of work are expected to facilitate improved conditions for the optimal extraction of select cannabinoids using scCO_2_, which holds promise in the development of well-characterized medicinal cannabis formulations. As to our best knowledge, this is the first study to report the uHPLC quantification method for the analysis of 11 cannabinoids from scCO_2_ extract in a single run with more than 1 min peak separation.

## 1. Introduction

Cannabis is considered a highly promising medicinal plant due to its purported array of therapeutic properties, although according to a recent survey it is most commonly used as an illicit drug [1]. It contains a variety of phytochemicals (around 500 compounds) including sugars, cannabinoids, alkaloids, phenolic compounds, and terpenes [2]. However, the psychoactive and psychotropic property of cannabis is particularly related to the presence of cannabinoids. In medicinal cannabis, cannabidiol (CBD), and tetrahydrocannabinol (THC) are most commonly occurring cannabinoids. From an industrial point of view, CBD is currently considered the most valuable cannabinoid as it possesses a broad range of therapeutic properties, such as anticonvulsant, anxiolytic, neuroprotective, antibiotic, anti-inflammatory activity, and anti-oxidant [3,4]. That said cannabinol (CBN), cannabigerol (CBG), and cannabichromene (CBC) have also shown antifungal, antibacterial, anti-inflammatory, and analgesic properties [5].

The therapeutic properties in natural products are also due to the presence of various phytochemicals, such as glucosinolates, lignans, carotenoids, polyphenols, etc. [6]. Therefore, there is a growing interest to adopt the “natural” or alternative approaches to cure so-called lifestyle diseases, rather than using pharmacologic therapy. The use of natural products for the treatment or to prevent the diseases to gain the healthy lifestyle is progressing [7]. 

The supercritical fluid extraction (SFE) method has gained increasing interest as a means of extracting cannabinoids from cannabis due to its selective extraction, short processing time, low running cost, and low impact on the environment, compared to conventional solvent-based extraction methods. SFE is a process in which the supercritical fluid (SCF) separates or dissolves components from the plant matrix according to their solvating properties. The solvating property of extracting the component can be maintained by changing the temperature and pressure above the critical point. Therefore, due to the tunable nature of SCF it can only target the desired substance from the sample matrix [8]. Additionally, the design of the SCF extracting chamber also plays an important role in the interaction of SCF substance with a targeted analyte. Proper experimental design can also maximize the yield of the targeted component with high purity. Various theoretical and physical factors (such as inlet and outlet valves of SCF into the extracting chamber, and separating chamber) also participates simultaneously to obtain the high yield. Furthermore, managing the pressure and throttling effect of SCF during extraction collection can enhance the extractability of SCF [9,10].

After the extraction of cannabinoids, the fast and reliable quantification method is an essential step of the analysis. Gas chromatography (GC) is considered as the most useful quantifying and separating technique for the analysis of cannabinoids as it considers a simpler and faster technique compared to high-performance liquid chromatography (HPLC) [11]. However, during GC analysis, acidic cannabinoids convert into their neutral form due to the thermal effect. Therefore, the proper quantification of cannabinoids through GC derivatization step is necessary [12]. HPLC is also considered as the simplest method to analyze the cannabinoids from the cannabis plant and other matrixes, as it does not require high heating step for analysis of the cannabinoids. Therefore, previously a number of HPLC methods have been developed for the determination of cannabinoids [13].

Recent surveys have shown that cannabinoids quantification assays via HPLC focused on the analysis of main cannabinoids (THCA, THC, CBN, CBD, and CBDA) in a single run [14]. However, these methods were either not validated properly or unable to perform the efficient separation of cannabinoids [12,15,16]. Because of the complex nature of the plant extract, the major cannabinoids peaks overlap (such as, CBGA/CBN, and CBG/CBD), which affect the analysis [3].

Previously, a number of studies focused on the SCF conditions for the extraction of cannabinoids. However, the setup of SCF is equally important to gain a high yield of cannabinoids. Therefore, this study was aimed to develop a setup for the SCF extraction of cannabinoids with high yield at optimal operating conditions from cannabis plant material. In addition, reversed-phase uHPLC-DAD quantification assay was developed for the effective quantification of 11 main cannabinoids and their acids with good peak separation.

## 2. Material and Methods

### 2.1. Chemical and Reagents

Eleven cannabinoids, namely cannabidivann (CBDV), tetrahydrocannabivarin (THCV), cannabidiol (CBD) with 99.66% purity (Lot: FE08071702), cannabigerol (CBG) with 98.98% purity (Lot: FE06241604), cannabidiolic acid (CBDA) with 98.3% purity (Lot: FE12011601), cannabigerolic acid (CBGA), cannabinol (CBN) with 99.37% purity (Lot: FE06131701), delta 9-tetrahydrocannabinol (Δ9-THC) with 97.66% purity (Lot: FE1041701), delta 8-tetrahydrocannabinol (Δ8-THC), cannabichomene (CBC) with 97.60% purity (Lot: FE10011502), and delta 9-Tetrahydrocannabinol acid (THCA-A) with 99.18% purity (Lot: FE12121601) stock solution with the concentration of 1000 µg/mL in acetonitrile or methanol (acid forms) as reference standards were purchased from Cerilliant, a Sigma Aldrich company (Kinesis Australia Pty Ltd., Redland Bay, QLD, and Novachem Pty Lt, Victoria, Australia). All other solvents (methanol, acetonitrile, and phosphoric acid) were purchased from Merck.

### 2.2. Sample Collection

**Figure 1:**
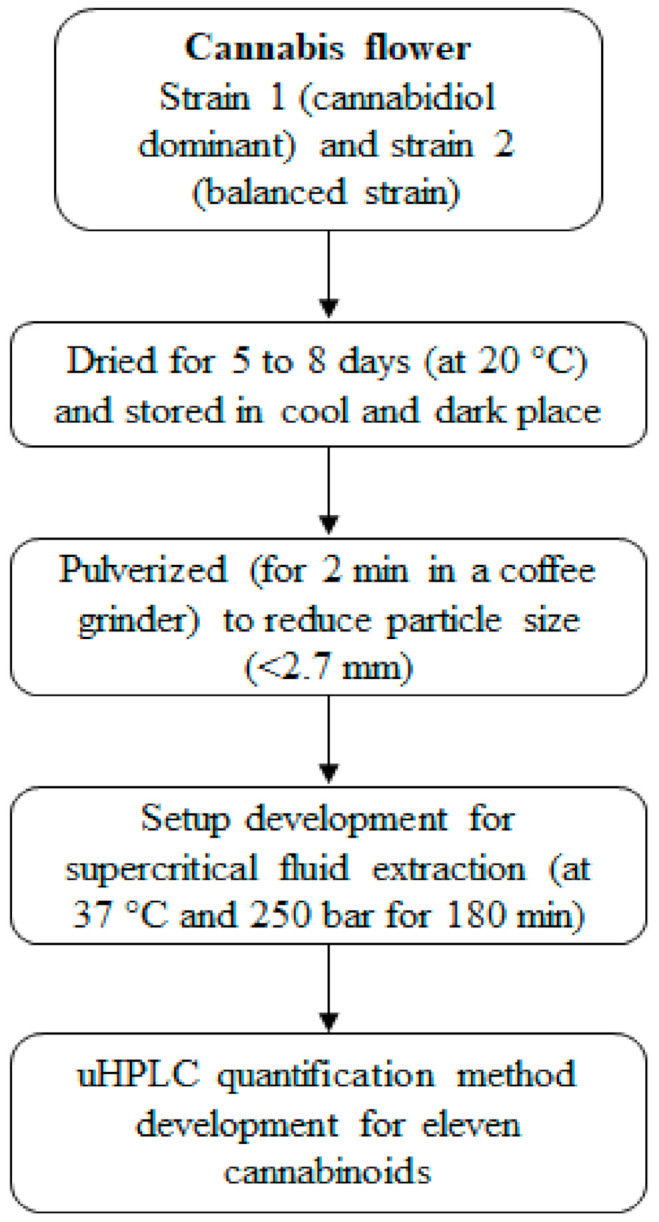
The schematic representation of the cannabis sample preparation and analysis.

Cannabis material has been obtained by the School of Pharmacy, The University of Queensland, under Queensland Health Approval license UNIR008335019; cannabis strain 1 (cannabidiol dominant; had <10% *w/w* of total cannabinoids and among them 90% *w/w* cannabinoids were CBD and CBDA, whereas, THC and THCA were around 5% *w/w*), and strain 2 flower material (had around 14% *w/w* total cannabinoids and among them ~50% *w/w* cannabinoids were cannabidiol and ~45% *w/w* cannabinoids were tetrahydrocannabinol, referred to as the ‘balanced strain’). These cannabis samples with Sativa genotype were planted on 4th May, 2017 under the best-growing conditions (12 to 18 h light exposure at 23 °C). Cannabis sample (from plant flowers) collected at the fluorescence stage and dried for 5 to 8 days at 20 °C (with total moisture <10%). This sample was pulverized for 2 min in a coffee grinder (Breville, model BCG200) to obtain a particle size of <2.7 mm. The schematic representation of the cannabis sample preparation and analysis is shown in Figure 1.

### 2.3. The Supercritical Fluid Extraction (SFE) and Setup Optimization

In this study, the supercritical fluid extraction (SFE) of cannabinoids was performed using scCO_2_. The density of CO_2_ plays an import role for the extraction of selective components from cannabis. Previously, Span and Wagner [15] studied the thermodynamic properties of CO_2_ at various states and found that at 250 bar and 37 °C it attained a high density (893.7 kg/m3) in a supercritical state. That is why this study was performed at 250 bar and 37 °C for 3 h to obtain a better extraction of cannabinoids from cannabis. Furthermore, two different SFE operating systems were used for studies to evaluate the optimal set-up for extraction.

### 2.4. Nottingham Unit

The SFE Nottingham unit (Teledyne ISCO, D-series) was used for the experimental designs A to D. The assembly of Nottingham unit was based on the liquid CO_2_ cylinder and syringe compressor to convert into scCO_2_. However, one main upgrade of the experimental design was the sample holding chamber, and their inlet and outlet positions for CO_2_ were designs in the lab. This demonstrates the uniqueness of the designed experimental setup and has led to novel extraction results, which are not reported in literature in the authors’ best knowledge. The maximum sample holding capacity of SFE extraction stainless steel vessel for Nottingham unit was 60.0 mL. The syringe pump attached to the liquid CO_2_ cylinder can hold up to 250 mL of liquid CO_2_ (60 bar) and regulate the desired pressure in extracting chamber. Glass wool and a stainless-steel filter was used on the top of sample holding reactor to separate the extract from grinded plant material.

#### 2.4.1. Experimental Setup A

**Figure 2:**
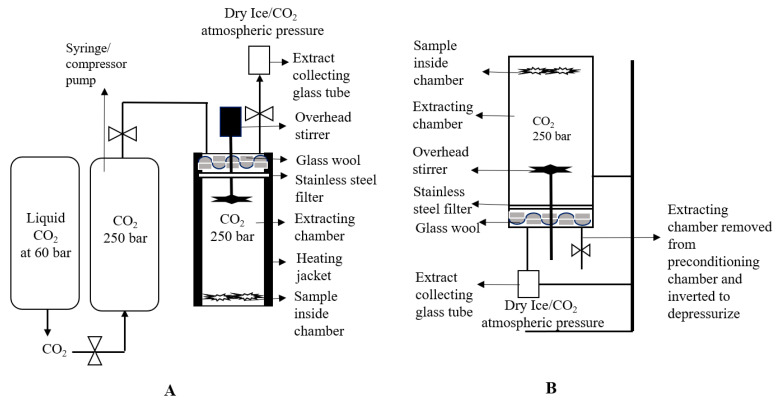
Schematic representation of the SFE Nottingham unit for the experimental setup A (**A**); Sample containing chamber was inverted and placed on stirrer for the depressurization (**B**).

The 1.0 g sample of cannabis strain 1 was placed inside the extracting chamber and filled with CO_2_. The temperature of the extracting chamber was controlled by using a heating jacket to obtain the desired density of CO_2_ in sub or supercritical state. The liquid CO_2_ dissolved the matrix from the sample according to its density. An overhead stirrer was also used (200 rpm) to help the proper dissolution of a matrix (as represented in Figure 2A). After extraction, the extracting vessel was removed from the SFE extraction system and reverted into collecting vessel to acquire a maximum amount of extracting material with the help of gravity, as shown in Figure 2B.

#### 2.4.2. Experimental Setup B

To increase the yield of cannabinoids from the cannabis sample by using SFE, the extraction chamber was rewashed with fresh CO_2_. In this procedure, the sample containing chamber was firstly filled with CO_2_ (at 250 bar, 37 °C) for 3 h to complete the extraction. After extraction, it was depressurized from 250 bar to 100 bar in the extract collecting chamber, as shown in Figure 2, and then the pressure was maintained again at 250 bar with fresh CO_2_ through a pressure regulating syringe. After 30 min, the sample holding chamber was disconnected from a back pressure regulator and again depressurize to 100 bar. After collecting the extract, the sample holding chamber was refilled again with fresh CO_2_ at 250 bar to avoid the super-saturation of CO_2_ from cannabinoids. This extracting chamber was finally fully depressurized at 0 bar after 30 min of extraction. The safety valve was removed and washed with methanol (5 mL) to collect the extract stuck to it. 

#### 2.4.3. Experimental Setup C

In this experimental design, the extraction of cannabinoids was also performed at 250 bar and 37 °C for 3 h. However, the depressurization or washing of the sample after extraction was performed three times. In this process, after 3 h of extraction, the sample holding chamber was removed from the back pressure regulating syringe pump and inverted into a sample collecting chamber with the help of the stand, as presented in Figure 2B. The sample extracting chamber was fully depressurized at 0 bar. After depressurization, the safety valve was removed and washed with 5 mL methanol to obtain the extract if it stuck to the valve. This sample holding chamber was attached again to the pressure regulating syringe pump. The pressure and temperature were maintained again at 250 bar and 37 °C. After 30 min of extraction, depressurization was performed again. The safety valve was also removed and washed with methanol as there was no pressure inside the chamber. After refilling CO_2_, full depressurization of the sample holding chamber and washing of the rod with methanol was repeated similarly again to maximize the yield of the cannabinoids.

#### 2.4.4. Experimental Setup D

In this procedure the operating conditions of extraction were similar (250 bar, 37 °C, 3 h) as performed in earlier experiments. However, the glass wool was not used and depressurization of sample holding chamber was performed in an upward direction (as shown in Figure 2A). The full depressurization (at 250 bar to 0 bar) was acquired after 30 min of CO_2_ refilling. Overall, rewashing of the sample was performed three times (first after 3 h, second after 30 min and third after 30 min). After complete extraction, the safety valve was opened and washed with 5 mL methanol.

### 2.5. Helix Unit

**Figure 3:**
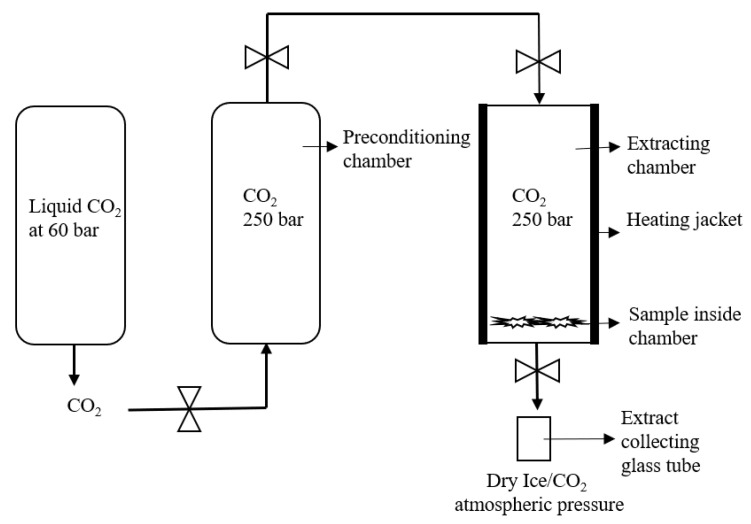
Schematic representation of the Helix unit for setup E extraction.

The SFE Helix unit (applied separations) was used for the experimental setup B. The maximum sample holding capacity of the helix stainless steel sample holding chamber was 100 mL. The desired internal temperature was monitored by the heating jacket. For the Helix unit, the maximum operating temperature and pressure were 60 °C and 700 bar. The back pressure was directly regulated by the preconditioning chamber from the liquid CO_2_ cylinder, as shown in Figure 3.

In this experiment, the SFE of the cannabis sample was performed at 250 bar and 37 °C for 3 h. The 1 gm grinded cannabis flowers sample strain 1 was placed on the bottom of the chamber. The CO_2_ stream was entered from the bottom inlet of the chamber and extraction was carried out for 3 h. In addition, there were two built-in filters on the bottom and top end of the sample holding cylinders to separate the plant material and CO_2_ from the chamber. After extraction, the CO_2_ with the dissolved matrix entered into the separating chamber, where the pressure was around 50 bar to avoid the throttling effect of dry ice (as represented in Figure 3). The extract was collected in the sample collecting vessel, attached to the bottom of the separating chamber. The weight of the extract collecting vessel was measured before and after extraction to estimate the yield of the extract. The sample was washed with the continuous flow of CO_2_ for 10 min and a dry sample was collected in the sample collecting vessel.

The SFE Helix unit (applied separations) was used for the experimental setup E to G. Similarly to the assembly of the Nottingham unit, the Helix unit was originally based on a liquid CO_2_ cylinder and preconditioning chamber to convert into scCO_2_. However, the sample holding chamber and their inlet and outlet positions for CO_2_ were designs in the lab. The maximum sample holding capacity of the helix stainless steel sample holding chamber was 100 mL. The desired internal temperature was monitored by the heating jacket. For the Helix unit, maximum operating temperature and pressure was 60 °C and 700 bar. The back pressure was directly regulated by the preconditioning chamber from the liquid CO_2_ cylinder, as shown in Figure 3.

#### 2.5.1. Experimental Setups E and F

The SCF extraction without using a separating chamber was performed in two different methods. In the first method, the CO_2_ stream was entered from the top of the sample holding chamber (as illustrated in Figure 2). However, all the other experimental conditions were similar, as performed in setup E experiment. After 3 h of extraction, the extract was collected from the bottom of the sample extracting chamber in a collection chamber. The weight of extract collecting vessel was measured before and after extraction to estimate the yield of extract.

#### 2.5.2. Experimental Setup G

**Figure 4:**
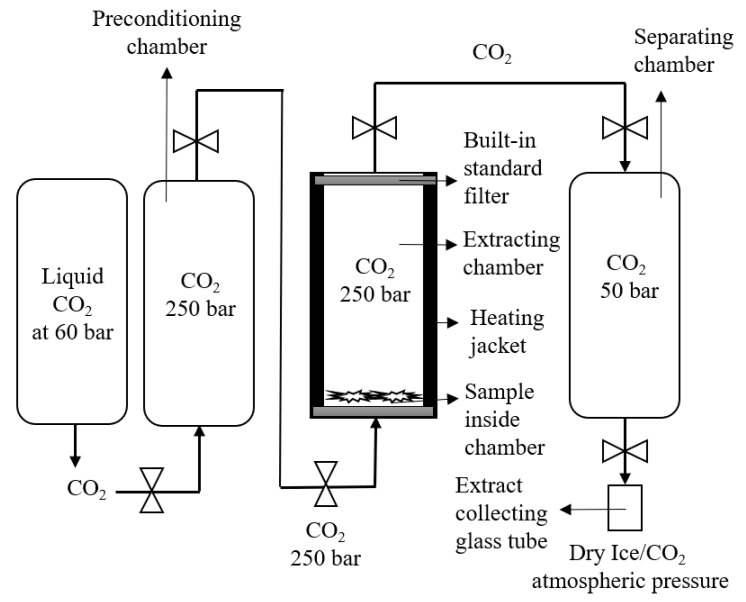
Schematic representation of the Helix unit for setup B extraction.

In this experiment, the SFE of the cannabis sample was performed at 250 bar and 37 °C for 3 h. The 1 gm ground cannabis sample was placed on the bottom of the chamber. The CO_2_ stream was entered from the bottom inlet of chamber and extraction was carried out for 3 h. In addition, there were two built-in filters on the bottom and top end of the sample holding cylinders to separate the plant material and CO_2_ from the chamber. After extraction, the CO_2_ with the dissolved matrix entered into the separating chamber (as shown in Figure 4), where the pressure was around 50 bar to avoid the throttling effect dry ice. The extract was collected in the sample collecting vessel, attached to the bottom of the separating chamber. The sample was washed with the continuous flow of CO_2_ for 10 min and the extract was collected in the sample collecting vessel.

### 2.6. uHPLC-DAD Quantification

The uHPLC method was initially developed by Shimadzu Scientific Instruments and transferred to PACE with a loan of a Prominence-I LC-2030 C3D liquid chromatography unit, with a Shim-pack XR ODS-II (2.20 µm, 3.0 mm ID × 75 mm).

#### 2.6.1. Standard Solution Preparation

The concentrated solution of each cannabinoid standard (1000 µg/mL) was diluted in methanol to make 250 µg/mL as a stock solution. The calibration curve of mixed standards with each cannabinoid at 1.0 to 25.0 µg/mL concentrations was prepared in methanol by using the cannabinoids stock solution. All standards solutions were stored at −80 °C.

#### 2.6.2. Instrumentation

Shim-pack XR-ODSII, spherical silica particles, 2.2 µm particle size (Shimadzu Scientific) reversed-phase C18 chromatographic column was used for the separation of cannabinoids. The quantitation method was standardized using Lab Solutions software or Cannabis Analyser (Shimadzu Scientific Instruments, Sydney, NSW, Australia).

#### 2.6.3. Mobile Phase Elution Program

The mobile phase A was the mixture of MilliQ water and phosphoric acid (millimolar; mM) (0.07% H_3_PO_4_/99.93% MilliQ H_2_O; adjusted to pH between 2.22 to 2.26). Mobile phase B was the mixture of methanol and phosphoric acid (mM; 0.07% H_3_PO_4_/99.92% methanol; adjusted to pH 2.43 to 2.48). The column oven temperature was maintained at 50 °C and the flow rate was 1.0 mL/min to maintain the column pressure (~5400 to 5600 psi). The volume of injection was 10 µL and the total runtime was 45 min. Initially, mobile phase B (*v/v*) was adjusted at 65% for 1 min. Then, the percentage of mobile phase B was gradually increased from 65% to 72% over the 25 min time period. After that, it finally increases to 95% during a 5 min time period. After maintaining these conditions for 2 min the initial ratio of mobile phases was adjusted and re-equilibrated the column for 12 min.

### 2.7. uHPLC Method Validation

#### 2.7.1. Selectivity/Identification

To identify the specific cannabinoid in a mixture, the retention time of each standard cannabinoid was ensured separately. For this purpose, the complete UV-visible spectra of each cannabinoid were recorded and compared with the retention time of the mixture.

#### 2.7.2. Precision and Accuracy

The accuracy, repeatability, and intermediate precession of the method were determined by preparing the three samples of each low medium and high concentration (2.5, 10, and 20 µg/mL) of 11 cannabinoids standard mixture on inter and intra days. 

#### 2.7.3. Linearity

The linearity of the method was demonstrated by preparing seven different concentrations of standard mixture solution in methanol (containing 11 cannabinoids) from 1.0 µg/mL to 25 µg/mL. The selected range of calibration curves was plotted in triplicates on three consecutive days. The regression of the coefficient (*r^2^*-value) of each calibration curve was calculated to determine the linearity of the method.

#### 2.7.4. Limit of Detection (LOD) and Limit of Quantitation (LOQ)

LOD and LOQ were analyzed by plotting a calibration curve within the range of seven different non-zero detection limit (DL) and quantitation limit (QL) values. The DL and QL of all 11 cannabinoids were calculated by using the following formulas:

(1) $$DL=\frac{3.3\;\sigma }{S},$$

(2) $$QL=\frac{10\;\sigma }{S},$$

where σ = response of standard deviation and S = slope of the calibration curve.

### 2.8. Statistical Analysis 

All analyses were performed in triplicates. The statistical analysis was obtained by using Minitab 17 software.

## 3. Result and Discussion

Due to the complexity and lack of knowledge of SFE factors interactions and in-depth fluid dynamics, SFE is considered a black box design. However, by exploring different experimental parameters, extraction principles and detailed point-to-point process information can produce favorable results [8]. Optimization of the setup to obtain fruitful results is the first stage of every experimental design. Therefore, this study was conducted to design the best setup for the extraction of cannabinoids from cannabis by using SFE. For this study, two different units of SFE were used for the extraction, the Nottingham unit, and the Helix unit. The results are represented in Table 1.

### 3.1. Nottingham Unit

**Figure 5:**
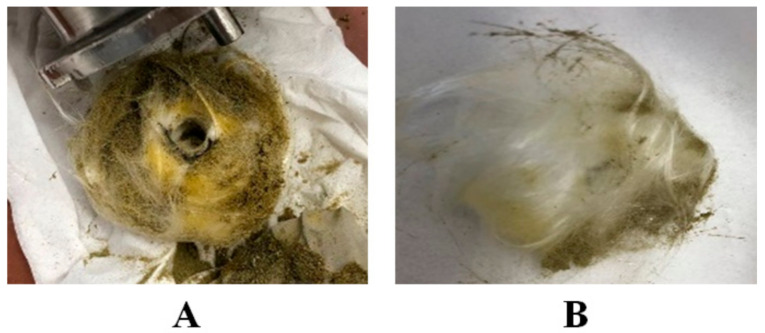
Schematic representation of glass wool after experimental setup A (**A**) and SFE Nottingham unit extraction (**B**).

The Nottingham unit was used for experimental setup A and the performed conditions are presented in Table 1. The extraction of cannabinoids from the cannabis sample occurred at 37 °C and 250 bar. These conditions were selected to obtain the maximum density of CO_2_. Recent studies only focused on the temperature and pressure of CO_2_ for SFE. It was reported that the adoption of very high pressure (up to 500 bar) decreases the selectivity of the cannabis extract [16], because above 250 bar the vapor pressure of the solute also increases. That is why at high pressures, high temperature has a greater influence on the solubility than the density [17]. Therefore, in this study, a carefully low temperature was used to increase the mass transfer rate of cannabinoids from plant to CO_2_.In experimental setup A, the glass wool and stainless-steel filter was used for the proper separation of cannabinoids scCO_2_ extract from original plant material. However, during the depressurization of the scCO_2_ extract (from 250 bar to atmospheric pressure) it was found that all extracted material (oil) was soaked in glass wool and the yield was quite low (as represented in Figure 5A). To avoid the extract soaking in glass wool and to improve the extraction, experimental setup B was designed.

In experimental setup B, a comparatively small amount of glass wool and 2 stainless steel filters were used to avoid the contamination of the extract from original ground material. Additionally, the depressurization was performed in triplicates (first; from 250 bar to 100 bar, second; after 30 min extraction with fresh CO_2_, from 250 bar to 100 bar, third; again, filled up with fresh CO_2_ for 30 min and fully depressurized from 250 bar to 0 bar). As a result, a sharp increase in the total yield of the extract was observed in experimental setup B (127.67 mg) as compared to experimental setup A (26.70 mg). Furthermore, the soaking of extract in glass wool also decreases markedly (Figure 5B).

After the inspiration of experimental set-up B results, experimental setup C was designed, in which full depressurization from 250 bar to 0 bar was performed in triplicates. From the results, it was shown that the total yield and the % of cannabinoids were increased two-fold as compared to experimental set-up B (as shown in Table 1). However, the two main issues were observed. Contamination with original material was not fully resolved and high leakage of cannabinoids on the safety valve and glass wool was found.

To resolve these main issues, the glass wool was fully removed in experimental set-up D and the experiment was performed only using stainless steel filters. However, from the results, it was cleared that the total yield decreased sharply as compared to experimental setups B and C. That is why a further study was performed on Helix unit of SFE as it has built-in filters and contamination chances with original plant material were almost zero or very low. 

#### Helix Unit

**Figure 6:**
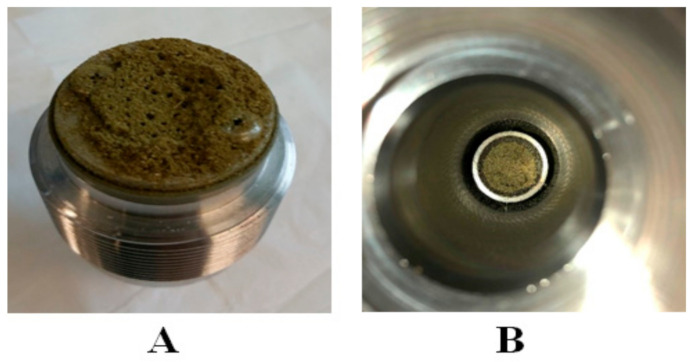
Schematic representation of bottom floor of the sample holding chamber in Helix unit. (**A**): bottom filter was blocked during depressurization of CO_2_ from bottom outlet. (**B**): cannabis sample placed at the top of cone.

After Nottingham, the Helix unit has been used to obtain the high yield of scCO_2_ extract with a high amount of cannabinoids from cannabis. For this purpose, experimental setups E to G were designed, as shown in Figure 3 and Figure 4. Experimental setup E was very simple because the built-in stainless filter was placed on both ends of the sample holding the chamber. The CO_2_ was entered inside the sample holding chamber from the top inlet valve and the sample was placed in a stainless steel cone in the bottom of the sample holding chamber (Figure 6B). However, after the depressurization from the bottom inlet, it was found that the stainless steel filter gets blocked and the plant material stuck on the filter, as illustrated in Figure 6A. Therefore, the extract was not collected. 

**Figure 7:**
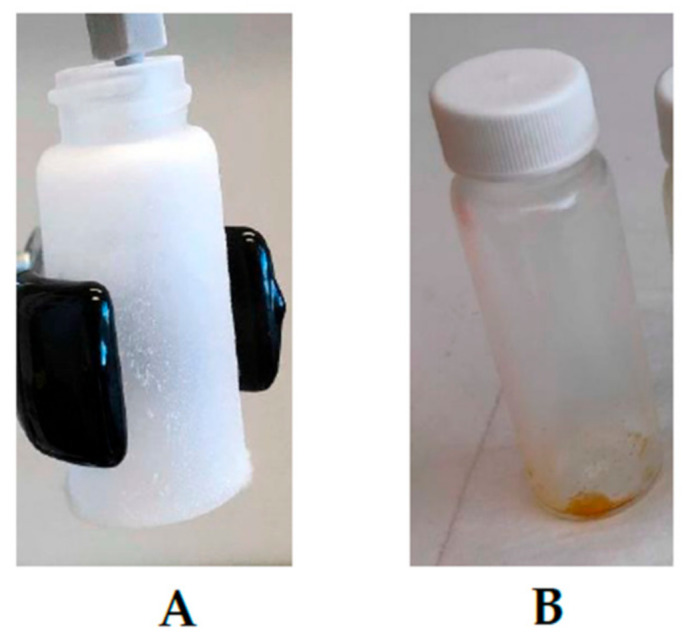
Throttling effect during the extract collection, (**A**): dry ice formation at sudden drop in pressure (250 bar to 0 bar), (**B**): Collection of extract at low pressure drop (50 bar to 0 bar).

To figure out this issue, experimental setup F was designed, in which the sample was also placed on the stainless steel cone in the bottom of the sample holding chamber. However, the stream of CO_2_ entered from the bottom inlet and depressurization from the top outlet (Figure 4). After the depressurization, it was observed that due to the sudden drop in pressure (250 bar to 0 bar), the throttling process occurred, as presented in Figure 7. As a result, the total yield of scCO_2_ extract was very low (14.20 mg, Table 1).

Therefore, to improve the setup of SFE extraction with Helix unit, experimental setup F was designed, un which after the sample holding chamber (maximum size 100 mL), the low-pressure regulating chamber/separating chamber (maximum holding pressure 100 bar) was adjusted. As a result, the good yield (53.92 mg, Table 1) was obtained after single depressurization.

### 3.2. Ultra-High-Performance Liquid Chromatography Coupled with DAD (uHPLC-DAD)

**Figure 8:**
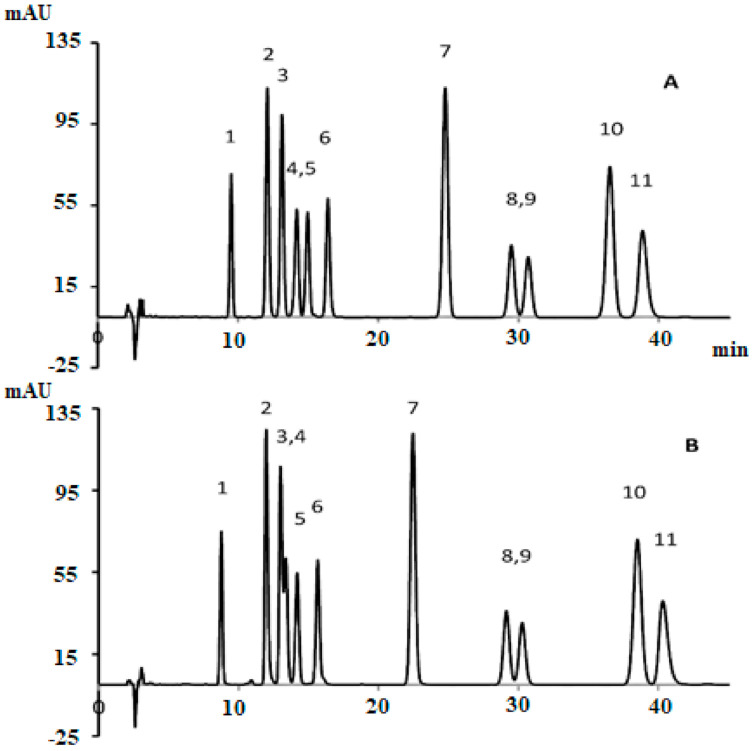
HPLC profile of 11 cannabinoids using an analytical column, (**A**) ACE 5 C18-AR (250 mm × 4.6 mm ID, 5 μm) and (**B**) conventional c-18 column [20]. Elution order: 1-CBDV, 2-CBDA, 3-CBGA, 4-CBG, 5-CBD, 6-THCV, 7-CBN, 8-d9THC, 10-CBC, and 11-THCA. Reprinted with permission from Ref. [20]. Copyright 2021 Copyright Ciolino.

uHPLC with DAD is considered as the most accurate and simple method for the quantification of cannabinoids. Because it is easy to perform on a routine basis, it efficiently separates the analyte and does not degrade the sample during quantification [18]. Several studies developed methods for the quantification of cannabinoids. Such as, De Backer, Benjamin [19] designed and validated a method to analyze the eight cannabinoids by using three chemotypes (including, fiber-type, intermediate-type, and drug-type) extracts (chloroform: methanol; 1:9 *v/v*) of the cannabis plant. Additionally, this method was validated with a clearly separated HPLC profile. In another study Ciolino, Ranieri [20] developed a new HPLC-DAD quantitation method to determine the 11 cannabinoids in cannabis samples by using two different columns. The analytical column, ACE 5 C18-AR (250 mm × 4.6 mm ID, 5 μm) gives better separation than the conventional c-18 column. The isocratic mobile phase system for ACE 5 C18-AR and Luna C-18 was 34: 66 and 26:74 for 0.5% acetic acid: acetonitrile. The total run time was 50 min and the figures of chromatogram are shown in Figure 8.

However, only five cannabinoid compounds were validated (CBN, THCA, CBDA, d9THCA, and CBD). These cannabinoids were also scrutinized in cannabis oils, extracts, plant, and their commercial products. In different states (free-flowing liquids or viscous compounds, semisolids, solids, emulsions, dispersions, aqueous and non-aqueous solutions) and polarities such as polar foodstuffs (beverages and sugary foods) nonpolar products (butter, balms/certain ointments), and substances with intermediate polarities (oral supplements and many topical foods). 

**Figure 9:**
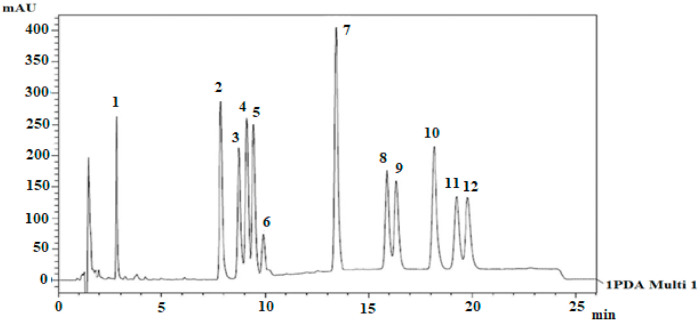
HPLC profile of 11 cannabinoids by using Luna C-18 analytical column [21]. Elution order: 1-I.S, 2-CBDA, 3-CBGA, 4-CBG, 5-CBD, 6-THCV, 7-CBN, 8-d9THC, 9-d8THC, 10-CBL, 11-CBC, and 12-THCA. Reprinted with permission from ref. [21]. Copyright 2021 Copyright Gul.

HPLC analysis of 11 cannabinoids cannabis extract and biomass was also performed by Gul, Gul [21]. The mobile phase system was gradient (water and acetonitrile with 0.1% formic acid). The separation chromatogram was obtained by using Luna C-18 column at 220 nm in 22.2 min run time. The elution order is also represented in Figure 9. This is quite similar to Figure 8 but overall, the efficiency of the separated peaks was low. Whereas, this method was validated for all selected cannabinoids and their concentration was also measured in 13 various samples of cannabis.

McPartland, MacDonald [22] used reversed-phase HPLC to investigate the binding affinity of THC and its acidic precursor THCA-A with CB_1_ and CB_2_ receptors in humans. In this method, the C18 column was used with a linear gradient mobile phase system and the chromatogram was obtained within 25 min. However, this study only focused on the stability of non-psychoactive cannabinoids (THCA-A) and their binding capability in human body receptors. However, the study revealed a greater binding affinity of THC CB_1_ (62-fold) and CB_2_ (125-fold) as compared THCA-A. 

Various other studies developed methods to determine six to seven main cannabinoids and their acidic precursors by using HPLC. However, these methods are not validated. Such as Romano and Hazekamp [23] had been used preheated cannabis (with 19% THC) extracts in olive oil, olive oil with water, ethanol, petroleum ether naphtha for the quantification of cannabinoids through HPLC.

**Figure 10:**
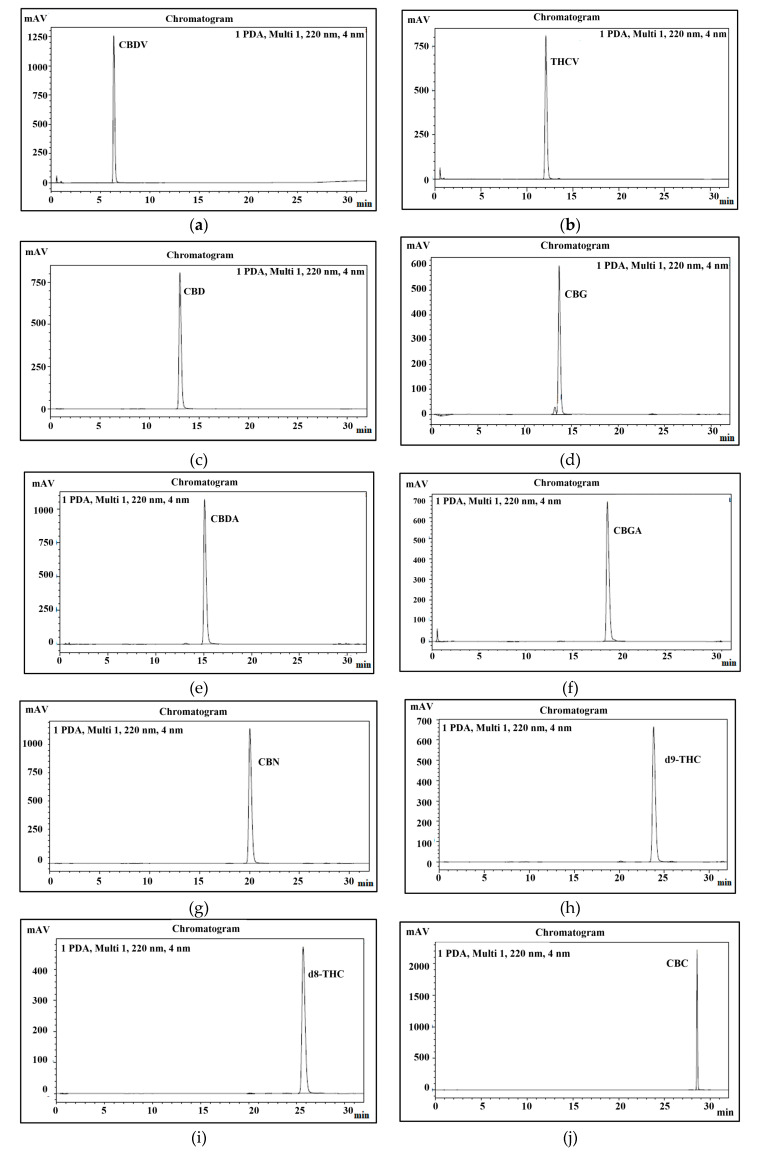

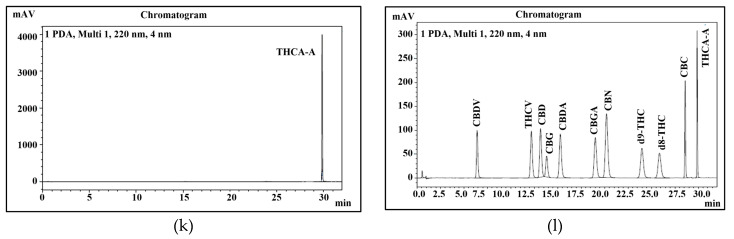
Peaks identification of eleven cannabinoids. (**a**) uHPLC profile of CBDV; (**b**) uHPLC profile of THCV; (**c**) uHPLC profile of CBD; (**d**) uHPLC profile of CBG; (**e**) uHPLC profile of CBDA; (**f**) uHPLC profile of CBGA; (**g**) uHPLC profile of CBN; (**h**) uHPLC profile of Δ^9^-THC; (**i**) uHPLC profile of Δ^8^-THC; (**j**) uHPLC profile of CBC; (**k**) uHPLC profile of THCA-A; (**l**) uHPLC profile of 11 cannabinoid mixture.

Therefore, this study aimed to develop a new method for the quantification of 11 main cannabinoids in cannabis and its derived products with good peak separation. In this study, psychoactive and non- psychoactive neutral cannabinoids and their acidic form were separated in 32 min (Figure 10). 

### 3.3. Method Validation

#### 3.3.1. Selectivity/Identification

The peaks of all 11 cannabinoids were fully separated during 32 min of program run. The retention time of each cannabinoid is shown in Table 2 and presented in Figure 10. To identify each peak of cannabinoid in a standard mixture, all cannabinoids were analyzed separately. Their elution order, retention time, and sensitivity were also confirmed through system suitability. It was also noticed that the pH of both mobile phases plays a very important role in stable separation and in maintaining a good retention time.

#### 3.3.2. Precision and Accuracy

The precision and accuracy of intra and inter days are represented in Table 3. The method for each cannabinoid was validated at three different levels of concentration, including low (2.5 µg/mL), medium (10 µg/mL), and high (20 µg/mL) as shown in Table 3. The %RSD of all selected 11 cannabinoids for the intra-day varied from 1.60% to 3.37% and for the inter-day from 0.20% to 1.75% respectively. Similarly, the variations in the accuracy level of each cannabinoid were also in an acceptable range. For the intra-day, the accuracy level for the low limit varied from 91.2 to 103.0 µg/mL, for the medium limit from 101.9 to 103.0 µg/mL, and the higher limit from 97.7 to 104.1 µg/mL. Whereas, for the inter-day, the accuracy level for the low limit varied from 88.26 to 99.6 µg/mL, for the medium limit from 101.12 to 103.04 µg/mL, and for the higher limit from 96.8 to 106.0 µg/mL. They are in the acceptable limit of 85.0 to 115.0% (±15%), except for LOQ 80.0 to 120.0% (±20%). 

#### 3.3.3. Linearity, the Limit of Detection (LOD), and Limit of Quantitation (LOQ)

The sensitivity of the method was obtained by determining the linearity, LOD, and LOQ (results are represented in Table 4). The obtained LOD of this analytical method ranged between 0.27 to 0.51 µg/mL, showing that a very low quantity of cannabinoids in extract can be measured by this method, without any guarantee of the imprecision or bias in the result of this assay. 

A calibration curve of 11 cannabinoid standard mixtures was performed to evaluate the concentration of cannabinoids in unknown samples or ground plant material. The calibration curve was conducted in triplicates on three consecutive days, with the stable, linear, and *r^2^*-value always >0.99 for each standard. Additionally, from the results of LOD (0.27 to 0.51 µg/mL) and LOQ (0.92 to 1.71 µg/mL), it was shown that the method was sensitive. The obtained LOD and LOQ values of cannabinoids were also comparable with previously developed methods [3,21].

### 3.4. Analysis of Cannabinoids

**Figure 11:**
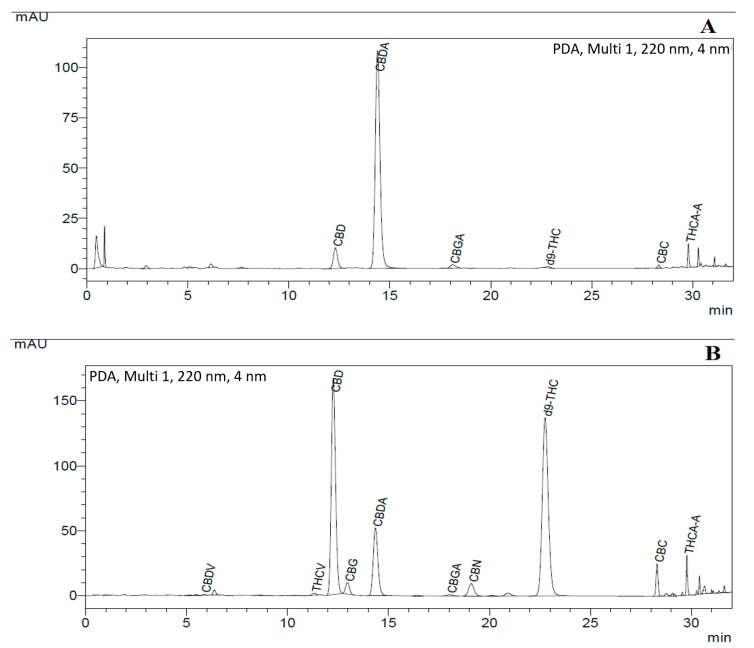
uHPLC profile of cannabis strains: (**A**) with dominant CBD + CBDA (around 90% *w/w*); and (**B**) with 55% *w/w* CBD+CBDA and 35% THC + THCA in the cannabinoid mixture.

Two different cannabis strains were used for the SCF extraction (as shown in Table 5) and their chromatogram is also represented in Figure 11, in which strain 1 was CBD + CBDA dominant (around 90% *w/w*) and strain 2 had an almost equal amount of CBD + CBDA (50% *w/w*) and THC+THCA (45% *w/w*) as compared to other cannabinoids. The results of well cannabis strains from cannabis strain extracts obtained from the final SFE setup are also presented in the table. 

## 4. Conclusions

The optimal setup configuration of supercritical fluid extraction (SFE) was achieved by systematically changing the inlet and outlet valve position of SFE for CO_2_ entrance and depressurization. However, scCO_2_ extraction conditions were fixed at 250 bar, 37 °C, 180 min, and 1 g plant material, to measure the cannabinoids yield during setup optimization. Additionally, the purity of the extract was also increased by using stainless steel built-in filters and an additional separating chamber. Furthermore, for the quantification of neutral cannabinoids and their acids, a highly sensitive reverse-phase uHPLC-UV-DAD method was developed. All selected cannabinoids showed good separation over the 32 min runtime (45 min with re-equilibration). Their relative retention time was also strongly influenced on the pH of mobile phases and the operating pressure of the column. The method was validated by analyzing the linearity, LOQ, LOD, accuracy, and precision in triplicates on inter and intra-day according to US-FDA guidelines. 

## Figures and Tables

**Table 1:** Conditions for SCF extraction of cannabinoids from cannabis.

Parameters	Set-Up A	Set-Up B	Set-Up C	Set-Up D	Set-Up E	Set-Up F	Set-Up G
Sample amount (g)	1	1	1	1	1	1	1
Pressure (bar)	250	250	250	250	250	250	250
Temperature (°C)	37	37	37	37	37	37	37
Density of CO_2_ (kg/m^3^)	893.7	893.7	893.7	893.7	893.7	893.7	893.7
Operating time (min)	180	240	240	240	180	180	180
Stainless steel filter	1	2	2	1	-	-	-
Glass wool amount (gm)	2	1	1	-	-	-	-
Stirring rate (rpm)	200	200	200	200	-	-	-
Number of depressurization	1	3	3	3	1	1	1
Extraction reactor size (mL)	60	60	60	60	100	100	100
Obtained yield (mg)	26.70	33.83 (after first depressurization)	61.82 from CO_2_ and 43.80 from rod (after first depressurization)	13.32 from CO_2_	-	14.20	53.92
		47.81 (after second depressurization)	69.02 from CO_2_ and 20.90 from rod (after second depressurization)	8.73 from CO_2_			
		46.03 (after third depressurization)	35.30 from CO_2_ and 11.70 from rod (after third depressurization)	9.52 from CO_2_ and 7.8 from rod			

**Table 2:** System suitability for higher concentration (20 µg/mL) of 11 cannabinoid standard mixtures.

Name of Cannabinoid	System Suitability (Peak Area ± SEM)	Retention Time (min) ± SEM
CBDV	656,922 ± 465	5.79 ± 0.018
THCV	899,571± 764	11.18 ± 0.036
CBD	963,188 ± 641	12.10 ± 0.045
CBG	400,828 ± 347	12.76 ± 0.050
CBDA	994,274 ± 804	14.15 ± 0.052
CBGA	996,755 ± 546	17.79 ± 0.071
CBN	1,641,927 ± 1305	18.87 ± 0.074
Δ^9^-THC	845,242 ± 948	22.56 ± 0.088
Δ^8^-THC	747,971 ± 711	24.41 ± 0.090
CBC	848,289 ± 708	28.25 ± 0.029
THCA-A	959,980 ± 1004	29.75 ± 0.017

**Table 3:** Precision and accuracy of 11 cannabinoids on intra-day and inter-day.

Name of Cannabinoid	Intra-Day				Inter-Day			
	Concentration (µg/mL)	Average Calculated Concentration (µg/mL)	Accuracy (%)	Precision (%) RSD	Concentration (µg/mL)	Average Calculated Concentration (µg/mL)	Accuracy (%)	Precision (%) RSD
CBDV	2.5	2.25	90.3	3.06	2.5	2.20	88.3	0.52
	10	10.29	102.9	2.04	10	10.19	101.9	0.66
	20	19.88	99.4	2.13	20	19.72	98.6	0.72
THCV	2.5	2.27	91.1	3.14	2.5	2.22	89.1	0.51
	10	10.29	103.0	2.03	10	10.18	101.8	0.72
	20	20.74	104.0	2.15	20	20.58	102.9	0.73
CBD	2.5	2.27	91.0	3.26	2.5	2.22	89.1	0.68
	10	10.18	101.9	2.47	10	10.11	101.1	0.64
	20	20.07	100.4	2.14	20	19.90	99.5	0.74
CBG	2.5	2.39	95.7	3.37	2.5	2.34	93.9	0.24
	10	10.29	103.0	2.45	10	10.21	102.1	0.65
	20	19.54	97.7	2.22	20	19.57	97.8	0.35
CBDA	2.5	2.43	97.3	2.27	2.5	2.39	95.7	0.63
	10	10.26	102.7	2.43	10	10.17	101.7	0.58
	20	19.87	99.4	1.86	20	19.73	98.7	0.67
CBGA	2.5	2.53	101.4	2.56	2.5	2.49	99.6	1.75
	10	10.23	102.4	1.90	10	10.18	101.8	0.57
	20	20.81	104.1	1.81	20	20.67	103.4	0.63
CBN	2.5	2.46	98.7	3.38	2.5	2.40	96.1	0.24
	10	10.30	103.	2.69	10	10.30	103.0	0.74
	20	20.96	104.8	2.14	20	21.20	106.0	0.71
Δ^9^-THC	2.5	2.37	95.1	3.90	2.5	2.31	92.4	0.43
	10	10.24	102.5	2.06	10	10.12	101.2	0.56
	20	20.82	104.1	2.36	20	20.63	103.2	0.81
Δ^8^-THC	2.5	2.52	100.7	3.15	2.5	2.46	98.5	0.20
	10	10.24	102.5	2.06	10	10.13	101.4	0.66
	20	20.76	103.8	2.20	20	20.93	104.7	0.72
CBC	2.5	2.48	99.4	2.93	2.5	2.42	96.9	1.56
	10	10.22	102.2	2.01	10	10.13	101.4	0.65
	20	19.48	97.4	1.94	20	19.35	96.8	0.63
THCA-A	2.5	2.43	97.3	3.21	2.5	2.37	95.1	0.64
	10	10.26	102.6	1.60	10	10.22	102.2	0.65
	20	20.08	100.4	1.83	20	19.97	99.9	0.48

**Table 4:** Linearity, the limit of detection (LOD), and limit of quantitation (LOQ).

Name of Cannabinoid	Linearity (r^2^)	LOD (µg/mL)	LOQ (µg/mL)
CBDV	0.992 ± 0.001	0.42	1.41
THCV	0.992 ± 0.001	0.33	1.11
CBD	0.995 ± 0.000	0.34	1.13
CBG	0.996 ± 0.001	0.31	1.03
CBDA	0.997 ± 0.000	0.32	1.08
CBGA	0.994 ± 0.001	0.32	1.06
CBN	0.993 ± 0.000	0.33	1.102
Δ^9^-THC	0.992 ± 0.001	0.37	1.26
Δ^8^-THC	0.995 ± 0.001	0.51	1.71
CBC	0.995 ± 0.001	0.29	0.99
THCA-A	0.993 ± 0.000	0.27	0.92

Where, Δ^9^-THC is *Delta-9-tetrahydrocannabinol* and Δ^8^-THC is *Delta-8-tetrahydrocannabinol*.

**Table 5:** Amount of cannabinoids in the SCF extract of two different cannabis strains.

Cannabinoids	CBD µg/mL (±S.E.M)	% CBD (*w/w*)	CBDA µg/mL (±S.E.M)	% CBDA (*w/w*)	THC µg/mL (±S.E.M)	% THC (*w/w*)	THCA µg/mL (±S.E.M)	% THCA (*w/w*)
Strain 1	92.23 ± 0.02	0.369	282.50 ± 0.13	1.130	9.48 ± 0.00	0.038	4.71 ± 0.01	0.019
Strain 2	252.72 ± 0.39	1.011	21.05 ± 0.25	0.084	197.50 ± 0.11	0.790	1.60 ± 0.00	0.006

## Data Availability

Not applicable.
